# An Experimental Investigation of Ultraweak Photon Emission from Adult Murine Neural Stem Cells

**DOI:** 10.1038/s41598-019-57352-4

**Published:** 2020-01-16

**Authors:** Tahereh Esmaeilpour, Esmaeil Fereydouni, Farzaneh Dehghani, Istvan Bókkon, Mohammad-Reza Panjehshahin, Noemi Császár-Nagy, Mehdi Ranjbar, Vahid Salari

**Affiliations:** 1 Department of Anatomical Sciences, School of Medicine, Shiraz University of Medical Sciences, Shiraz, Iran; 2 Histomorphometry and stereology Research center, School of Medicine, Shiraz University of Medical Sciences, Shiraz, Iran; 3 Psychoszomatic Outpatient Department, H-1037 Budapest, Hungary; 4 Vision Research Institute, Neuroscience and Consciousness Research Department, 25 Rita Street, Lowell, MA 01854 USA; 5 Department of Pharmacology, School of Medicine, Shiraz University of Medical Sciences, Shiraz, Iran; 6 National University of Public Services, Budapest, Hungary; 7 Department of Physics, Isfahan University of Technology, Isfahan, 84156-83111 Iran; 8 Department of Physical Chemistry, University of the Basque Country UPV/EHU, Apartado 644, 48080 Bilbao, Spain

**Keywords:** Neural stem cells, Biological physics

## Abstract

Neurons like other living cells may have ultraweak photon emission (UPE) during neuronal activity. This study is aimed to evaluate UPE from neural stem cells (NSC) during their serial passaging and differentiation. We also investigate whether the addition of silver nanoparticles (AgNPs) or enhancement of UPE (by AgNPs or mirror) affect the differentiation of NSC. In our method, neural stem and progenitor cells of subventricular zone (SVZ) are isolated and expanded using the neurosphere assay. The obtained dissociated cells allocated and cultivated into three groups: groups: I: cell (control), II: cell + mirror, and III: cell + AgNPs. After seven days, the primary neurospheres were counted and their mean number was obtained. Serial passages continuous up to sixth passages in the control group. Differentiation capacity of the resulting neurospheres were evaluated *in vitro* by immunocytochemistry techniques. Measurement of UPE was carried out by photomultiplier tube (PMT) in the following steps: at the end of primary culture, six serial cell passages of the control group, before and after of the differentiation for 5 minutes. The results show that neither mirror nor AgNPs affect on the neurosphere number. The UPE of the NSC in the sixth subculturing passage was significantly higher than in the primary passage (*P* < 0.05). AgNPs significantly increased the UPE of the NSC compared to the control group before and after the differentiation (*P* < 0.05). Also, the treatment with AgNPs increased 44% neuronal differentiation of the harvested NSCs. UPE of NSC after the differentiation was significantly lower than that before the differentiation in each groups, which is in appropriate to the cell numbers (*P* < 0.0001). The mirror did not significantly increase UPE, neither before nor after the differentiation of NSC. As a conclusion, NSC have UPE-properties and the intensity is increased by serial passaging that are significant in the sixth passage. The AgNPs increases the UPE intensity of NSC that pushes more differentiation of NSC to the neurons. The mirror was not effective in enhancement of UPE. As a result, UPE measurement may be suitable for assessing and studying the effects of nanoparticles in living cells and neurons.

## Introduction

## Neural stem cells

Stem cells are undifferentiated cells that are able to self-renew and give rise into specified cell types. It has long been thought that the mammalian brain is an organ with very little restorative power, but there has been a lot of evidence confirming the presence of endogenous stem cells in the central nervous system^1^. It is believed that the subventricular zone (SVZ) of the lateral ventricle and subgranular zone (SGZ) of dentate gyrus in the hippocampus contain stem cells where adult neurogenesis occurs. SVZ, the largest neurogenic region in the brain is extended from the olfactory bulb to the crossing of the anterior commissure. The existing neural progenitor cells generate neurons, astrocytes and oligodendrocytes *in vitro*^2–4^. NSCs reside in a vascular microenvironment that is rich in a group of substances, including peptide growth factors, classical and peptide neurotransmitters, and biogenic amines, which are required to regulate proliferation and differentiation of neural stem cells^5,6^.

## Ultraweak Photon Emission in Neurons

It has been evidenced that neurons like other living cells (e.g. plants, animals, and humans) have spontaneous ultraweak photon emission (UPE) through their metabolic reactions associated with physiological conditions^7^. The intensity of UPE varies from a few photons to several hundred photons per second per square centimeter, mainly with spectral range 200–800 nanometers^8^. In different scientific issues the terminology for UPE may be different such as biophotons, ultraweak emission, ultraweak bioluminescence, self-bioluminescent emission, photoluminescence, delayed luminescence, ultraweak luminescence, spontaneous chemiluminescence, ultraweak glow, biochemiluminescence, metabolic chemiluminescence, dark photobiochemistry, etc^9^. Since in our study the process of photon emission in the samples can be seen somehow as a weak delayed luminescence we prefer to use UPE instead the other terms. It has been shown that the origin of UPE is in direct connection with reactive oxygen species (ROS)^8,10,11^. The UPE intensity variations are associated with different physiological and pathological conditions, e.g. thermal, chemical and mechanical stress, mitochondrial respiratory chain, cell cycle and cancerous growth^12,13^. In fact, there is a direct correlation relationship between neural activity, oxidative reactions, EEG activity, cerebral blood flow, cerebral energy metabolism, and release of glutamate with UPE intensity^9,14–17^. Experiments demonstrated that cells can absorb UPE by photochemical processes and slowly release these photons as delayed luminescence^18–21^. It has been shown that the measurement of delayed luminescence emitted from the biological samples provide valid and predictive information about the functional status of biological systems^22^. On the other side, Popp proposed that biophotons (i.e. UPE) may present a wide variety of frequencies which originate from DNA^23^. He also found that biophotons are coherent and suggested that they may regulate life processes of an organism^24^. However, the coherence idea of UPE is under debate^25,26^ and it is still not clear that whether UPE is just a byproduct in biological metabolism or it has some informational or functional role.

## A Brief History

In 1967, Artem’ev *et al*.^27^ reported that electric pulses can induce nerve UPE (in the visible region of EM spectrum) in frog due to chemical reactions accompanying pulses, while a killed-neuron does not show any UPE. In 1984, Imaizumi *et al*.^28^ and in 1985 Suzuki *et al*.^29^ experimentally demonstrated that increased UPE can emerge after the induction of hypoxia states in the rat brain. In 1990, Karolyi^30^ measured weak bioluminescence of human subjects by two different methods. In 1995, Isojima *et al*.^14^ have shown that there is a correlation between the intensity of UPE and neural metabolic activity in the rat hippocampal slice. In 1997, Zhang *et al*.^31^ revealed that the intensity of UPE from intact brains isolated from chick embryos was higher than the medium in which the brain was immersed. In 1999, Kobayashi *et al*.^15^ detected spontaneous UPE in the rat’s cortex *in vivo* without adding any chemical agent or employing external excitation and found that the UPE correlates with the EEG activity, cerebral blood flow and hyperoxia, and the addition of glutamate increases UPE, which is mainly originated from the energy metabolism of the inner mitochondrial respiratory chain through the production of ROS. Kataoka *et al*.^32^ detected spontaneous UPE from cultured rat cerebellar granule neurons in the visible range and demonstrated that the UPE depends on the neuronal activity and cellular metabolism. Then, an intresting experimental discovery by Sun *et al*.^12^ revealed that UPE can be conducted along the neural fibers. In 2011, Wang *et al*.^33^ presented an *in vitro* experimental evidence about the existence of spontaneous UPE and visible light induced UPE (delayed luminescence) from freshly isolated rat’s whole eye, lens, vitreous humor, and retina. Then, in 2014 Tang and Dai^34,35^ provided experimental evidence that the glutamate-induced UPE can be transmitted along the axons and in neural circuits in mouse. Their approach has been recently simulated by Simon’s group^36,37^ at University of Calgary that optical communication in myelinated axons is possible from physics point of view. They have shown that neurons may act as biological optical fibers and UPE may have some informational role that it may even solve some cognitive open problems like binding problem^38^. Also, a recent controversial experiment in 2016 is the relevance of intelligence and UPE in the brain^17,39^. Despite different researches on neurons, there has not been published report on UPE from neural stem cells (NSCs) so far.

## The Aim of this Research

In this research, we first investigate UPE from murine NSCs and then study the UPE intensity in serial passaging. Then the effect of a mirror and nanoparticles on the increament of UPE intensity is investigated, and finally we study whether the variation of UPE intensity affects the differentiation of NSCs. Regarding the use of a mirror, we would like to see what happens if the emitted UPE is returned to the sample, i.e. Auto-optic effect^40^. Also, since there is growing interest regarding the use of nanoparticles (with unique physical and chemical properties) in diverse areas such as medicine (therapeutics and drug delivery), antimicrobial and anticancer agents, cosmetics, textiles, and electronics among others^41–44^, we also study UPE from NSCs that were exposed to silver nanoparticles (AgNPs). It has been evidenced that cells in the presence of AgNPs increased the UPE intensity and ROS production^45,46^. Here, we would like to investigate whether the presence of AgNPs affect the UPE intensity of NSCs.

## Materials and Methods

### Silver Nanoparticles (AgNPs)

**Figure 1:**
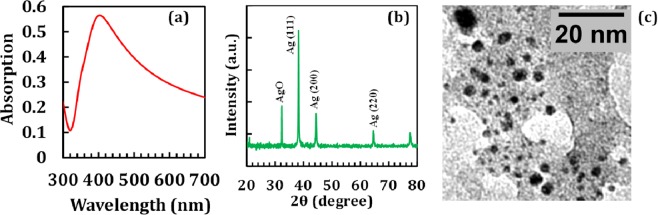
(**a**) Optical absorption spectrum, (**b**) XRD pattern and (**c**) TEM image of AgNPs.

AgNPs were synthesized by laser ablation from an Ag target (99.9% purity) in deionized water. The light source was an Nd:YAG pulsed laser with 1064 nm wavelength, 300 mJ energy per pulse, spot size of 3 mm^2^, fluence of about 10 J/cm^2^ and 5 ns pulse duration. The laser beam was focused normal to the target placed inside the 80 cc deionized water. The ablation proceeded for 40 min with 10 Hz repetition rate. Using inductively coupled plasma (ICP) analysis, the Ag concentration was obtained to be ≈15 ppm. Optical properties were measured in the 190–1100 nm range using a Lambda 25 spectrophotometer (Perkin Elmer). XRD was carried out using a Bruker D4 X-ray diffractometer. The Cu K (0.154 nm) X-ray line was used as the probe beam. The absorption spectrum of AgNPs (Fig. 1(a)) represents the characteristic plasmon absorption around 400 nm, characteristics of AgNPs with a beige color. Figure 1(b) shows the XRD pattern of AgNPs which indicates particles have crystalline structure. Figure 1(c) represents a typical TEM images of particles. From this image, the average particle size was estimated to be 2.4 nm.

However, instead of estimating the average NP-sizes, a proper size characterization should also be done in suspension, e.g. by using dynamic light scattering (DLS).

### Animals and experimental groups

**Figure 2:**
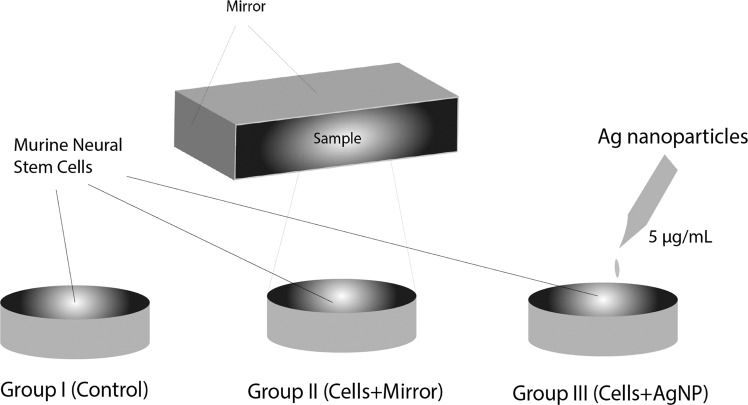
Three cell groups in the experiments: Group I: cells without any intervention as the control, Group II: cell + mirror, the cells first were placed in a small cubic mirror box and then measured after being in darkness, and Group III: cell + AgNPs, cells were incubated with 0.5 mL of AgNPs with a concentration of 5 *μ*g/mL for 24 hours. The cells were dissociated, centrifuged and resuspended in neurocult neural stem cell medium, counted and 10^5^ cells were transferred to 3 cm Petri dishes. The walls of the small cube box for group II are made up of normal home mirrors to investigate if UPEs are shifted back to the sample by mirror. For the group III the AgNPs were kept in a separate dark storage chamber at room temperature. In the all groups, each petri dish is placed 10 minutes in the dark box before starting the measurement to reduce the effect of delayed luminescence.

Five (5–8 weeks old) male C57-BL6 mice were obtained from Laboratory Animal Breeding Center, Shiraz University of Medical Sciences, were kept under standard conditions (12 hrs. light/12 hrs. dark, temperature 20–24 °C with free access to food and water ad libitum). All procedures performed in studies involving animals were in accordance with the ethical standards of Ethics Committee (i.e. ir.sums.rec.1394.s931) of the Shiraz University of Medical Sciences (SUMS), and the authors confirm that the experimental protocols were approved by SUMS and the above licensing committee. Isolated cells from SVZ region were cultured with complete medium and allocated into three groups (see Fig. 2): Group I: cells without any intervention as the control, Group II: cell + mirror, and Group III: cell + AgNPs, cells were incubated with 0.5 mL of AgNPs with a concentration of 5 *μ*g/mL for 24 hours. The walls of the cube box for group II are made up of normal home mirrors.

### Optimization of concentration, volume and time of incubation of AgNPs

**Figure 3:**
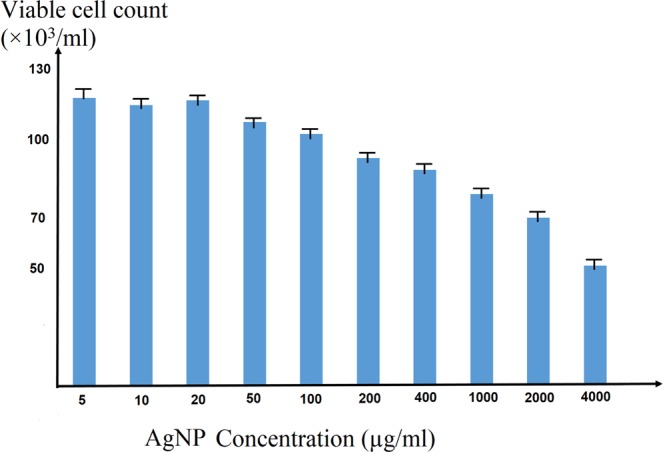
The viability of neural stem cells after the addition of AgNPs. The AgNPs are optimized in terms of their concentration and volume, with 0.5 mL of 10 different concentrations.

The AgNPs were kept in a separate dark storage chamber at room temperature. The initial concentration was estimated at 4000 mg/mL which was diluted for use at different concentrations. The nanoparticles were optimized in terms of their concentration and volume, with 0.5 mL of 10 different concentrations: 5, 10, 20, 50, 100, 200, 400, 1000, 2000, and 4000 *μ*g/mL (see Fig. 3), different volumes: 0.5, 1, 1.5 and 2 mL of nanoparticles which added to the petri dishes containing 10^5^ cells. After 24 hours of incubation, the cell viability and UPE were measured by trypan blue and PMT, respectively. Optimal concentration depends on the highest UPE (for 5 minutes) and viability rate. In order to optimize the incubation time, 0.5 mL of AgNPs at a concentration of 5 *μ*g/mL were added to cells at different periods of 12, 24, 36 and 48 hours. It is important to note that AgNPs in excess of 200 *μ*g/mL usually tend to suffocate/choke the cells rather then expressing their toxic potential. In addition, perhaps mass is not a good indicator for assessing such effects and one may consider a concentration (particles/mL of a given size class) as a better parameter^47^. In our analysis, the viability of murine neural stem cells at 4000 *μ*g/mL is surprisingly not zero, which is different with the previous studies on other samples.

### Neurosphere assay

A neurosphere is a free-floating cluster of neural and progenitor cells in a culture system and neurosphere assay provides a technique to isolate and differentiate neural stem and progenitor cells. It is an ideal clonal assay to quantitate the frequency of NSCs in a given heterogeneous cell population. For removing the brain, the mouse is anesthetized by intra-peritoneal injection of pentobarbital (120 mg/kg) and then underwent a cervical dislocation. The brain has been removed by peeling back the skull and frees the brain, rinsed with sterilized cold PBS to remove contaminations such as blood or hair. The SVZ was carefully removed microscopically from each brain and plated in neurosphere culture as previously described^48^. Briefly, SVZ of the both sides was removed, minced the tissue into very small pieces. The cell suspension was transferred into a sterile centrifugation tube, digested with pre-warmed 0.05% Trypsin-EDTA (Gibco, Carlsbad, CA) and incubated for 7 min at 37 °C. To stop the enzymatic reaction, soybean trypsin inhibitor (Sigma, St. Louis, MO) was added and centrifuged at 110 g for 5 minutes. The cell pellet was resuspended in neurocult neural stem cell (STEMCELL Technologies, Vancouver, Canada) medium and mechanically dissociated into single cells by repeated pipetting. The cell suspension was spun (110 g, 5 min) subsequently to remove the supernatant. The pellet were resuspended in complete neurosphere medium supplemented with EGF (20 ng/mL; STEMCELL Technologies), bFGF (10 ng/mL; STEMCELL Technologies) and heparin (2 *μ*g/mL; Sigma, St. Louis, MO), seeded into a 25-cm^2^ culture flask at a density of 5 × 10^5^ cells/mL and incubated in a humidied incubator with 5% CO_2_ for 7 days. During this period, cells proliferate to form neurosphere and ready for subculturing. The number of neurospheres was counted by an inverted microscope (Olympus, Center Valley, PA, USA). The mean for the number of neurospheres compared between all groups. To evaluate the capability of the resulting neurospheres for long-term serial passage, when the neurospheres became 150–200 *μ*m in diameter, enzymatically and mechanically were dissociated into single cells, replated in complete neurosphere medium supplemented with growth factors for 7 days. Six subculture passages were performed and their UPE measured by PMT. In order to significantly avoid the possibility of cellular electromagnetic communication we placed the culture flask into the incubator one by one or at distance.

### Differentiation assay and immunocytochemistry

The neurospheres of different groups derived from passage one to three were dissociated into single cells mechanically and enzymatically and seeded onto poly-L-ornithine coated 12-well plate at a density of 5 × 10^4^ cells/well in mouse NSC supplemented by 5% fetal calf serum (FCS) (Gibco), 20 ng/mL EGF, 10 ng/mL bFGF and 2 *μ*g/mL heparin for 3–4 days. When the cells reach 90% confluency, the medium was replaced by the growth factor free medium containing 5% FCS. After 4 to 6 days, the cells were fixed by paraformaldehyde (PFA) 4% for 10 min at room temperature for immunostaining of neuronal and astrocyte markers. The cells were washed 3X with PBS to remove PFA, incubated overnight at 4 °C with primary antibody neuronal marker, mouse monoclonal anti–III-tubulin (1:1000)(Promega, Madison, WI, USA), and glial marker, rabbit polyclonal anti-glial fibrillary acidic protein (GFAP; 1:500) (Dako Cytomation, Carpinteria, CA, USA) in PBST (PBS + 0.1% Triton-X) supplemented with 10% normal goat serum (NGS). After three washing with PBS, the cells incubated by the secondary antibody solution containing goat anti-mouse Alexa-Fluor 488 and goat anti-rabbit Alexa-Fluor 568 (1:500, Invitrogen) in PBS-Triton supplemented with 10% NGS for 45 minutes at room temperature in dark to detect the primary antibodies and Hoechst (1: 1000) for nuclear staining. A fluorescent microscope (Olympus IX-71) equipped with a Canon EOS digital Camera (Canon, Tokyo, Japan) was utilized to capture 10–15 representative pictures, to obtain merging images using Adobe Photoshop CS4 software. In 10 fields, the values of neurons and astrocytes were counted and the mean of each was calculated as a percentage.

### Viability rate

The cell sample was diluted 1: 1 (10 *μ*l of cell suspension and 10 *μ*l of trypan blue) with trypan blue (0.1% w/v in 0.15 M in PBS) and 10 *μ*l of the solution obtained was poured below the slurry. Then the Neubauer chamber was placed on the microscope stage and the viable cells were counted.

### Detection of UPE

Regarding the UPE measurements, the photomultiplier tube (PMT) is an extremely sensitive detector that can detect single photons, converting them to electrons to record the intensity in terms of time in the form of a graph. In our experiments we use PMT for UPE measurements. A photon counting system (Hamamatsu Photonics K.K., Electron Tube Center, Hamamatsu, Japan) was used to observe time-dependent photon emission intensity. The system is equipped with a R6095 PMT providing a maximum spectral response from 300 to 650 nm, cooled down to °C because the low power of our cooling system did not let us to cool it down more. Therefore, the numbers of counts in our experiments were low. The gate time for collecting the photon signal from the PMT was set at 1 second. The maximum detection of PMT is 420 nm with about 30% quantum efficiency in the range of 300 to 700 nm. The rise time for PMT was about 3 ns. By using an upper threshold we detected the number of counts in the dark box located in a dark room as 1 count per 5 minutes (c.p.5 min) at 1150 V, which was a relative dark count, i.e. subtracted dark current. In fact, we reduced the noise significantly by putting a threshold via PMT software to have a lower dark count. This caused a lower UPE counts for the samples, too. That is why we used the time intervals of 5 minutes to count the UPE in our study. The distance between the sample and the PMT lamp was selected at the shortest possible distance, 0.5 cm.

**Figure 4:**
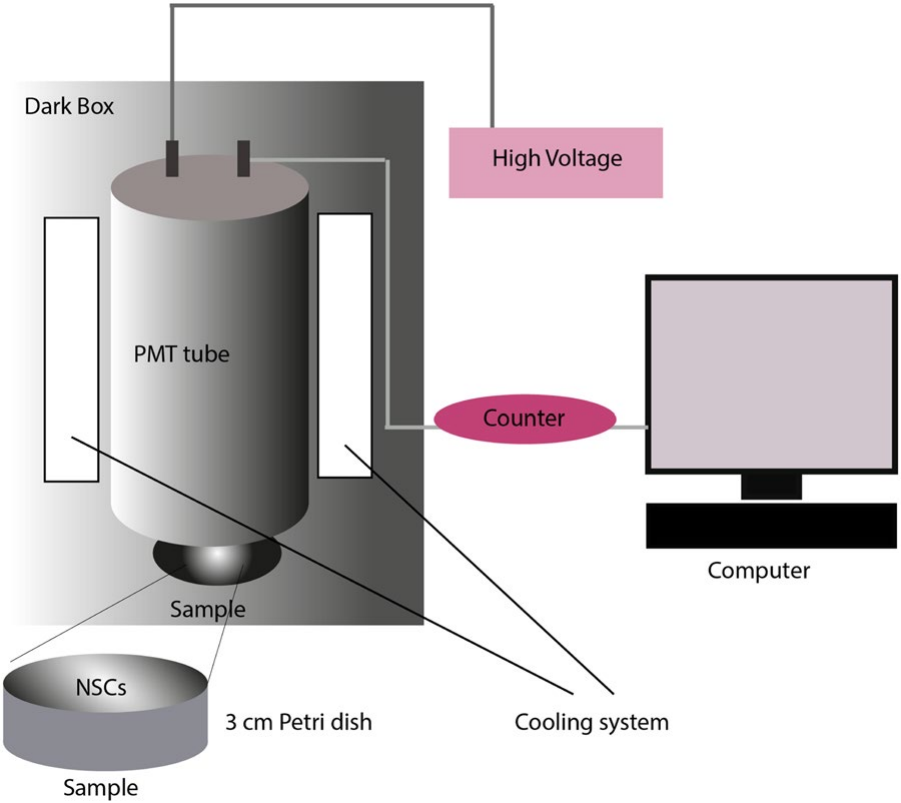
A schematic representation of the experimental setup for UPE measurement. The system is equipped with a R6095 PMT providing a maximum spectral response from 300 to 650 nm, cooled down to °C by cooling system. The PMT is connected to a counter, which is also connected to a computer for data to be digitally visible. The specimen was located in the dark room and the electronic equipments were placed outside the dark room, so that no other light except the cell UPE could be measured by the machine. First, the cells were transferred to 3 cm petri dishes, kept each petri dish 10 minutes in the dark box before starting the measurement, then placed under the PMT lamp in the main compartment and then measured for 5 minutes.

The PMT is connected to the G.G.104 (Parto-Tajhiz-Besat co - PTB) model counter, which is also connected to the laptop for data to be digitally visible. The specimen was located in the dark room and the electronic equipments were placed outside the dark room, so that no other light except the cell UPE could be measured by the machine (see Fig. 4). In each trial period, the medium’s emission and then the UPE of experimental groups were measured in a 5 minute period. The cells were dissociated, centrifuged and resuspended in neurocult neural stem cell medium, counted and 10^5^ cells were transferred to 3 cm Petri dishes which placed under the lamp in the main compartment and measured for 5 minutes. Then, we kept each petri dish 10 minutes in the dark box before starting the measurement. This helps to reduce a lot the possible delayed luminescence of the Petri dishes. Basically the delayed luminescence for the Petri dishes may take for few minutes. Measurement of UPE intensity by PMT was carried out in the following steps:

- At the end of primary culture in all three groups
- Six serial cell passages in control group
- At the beginning and the end of differentiation of neural stem cells.

All experiments were repeated three times.

#### Statistics analysis

Data analysis was performed using one-way ANOVA as appropriate (Prism 6; Graph pad Software Inc., San Diego, CA). Results were expressed as mean ± SE and the level of significance was set at a *P* value of less than 0.05.

### Ethical approval

All procedures performed in studies involving animals were in accordance with the ethical standards of Ethics Committee (ir.sums.rec.1394.s931), Shiraz University of Medical Sciences.

## Results

### Optimization of concentration, volume and time of incubation of AgNPs

In order to optimize the amount of AgNPs, various concentrations ranging from 5–4000 *μ*g/mL, various volumes: (0.5, 1, 1.5 and 2 mL), and various time period of incubation ranging 12–48 hours tested to the petri dishes containing 10^5^ cells. The optimum concentration, volume and incubation time of the AgNPs were 5 *μ*g/mL, 0.5 mL and 24 hours respectively, regarding the viability rate and enhancement of UPE.

### Neurosphere forming frequency

**Figure 5:**
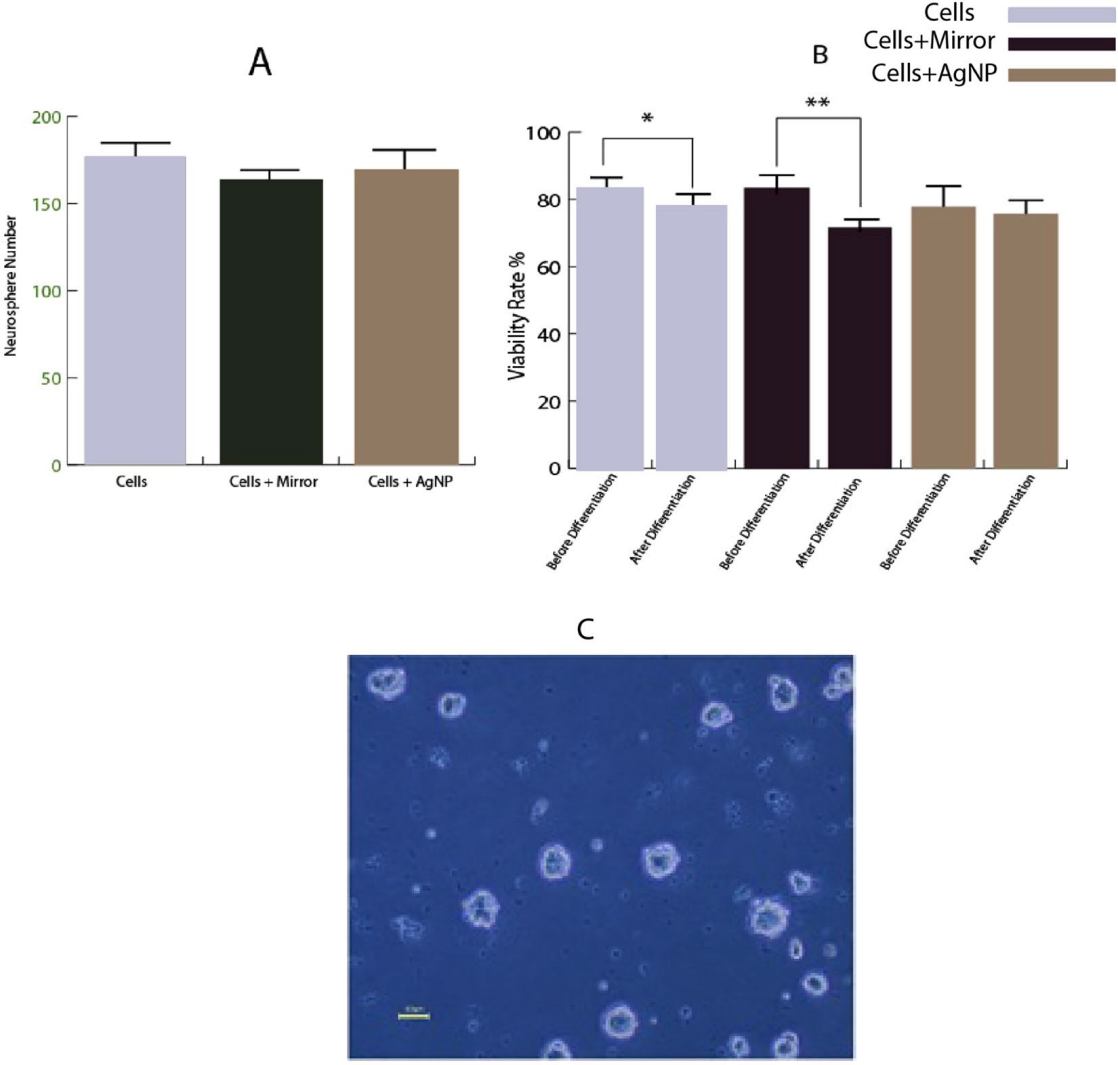
(**A**) Neurosphere formation quantitates the frequency of NSCs in a given heterogeneous cell population. Mean neurosphere forming frequency/condition after plating cells in culture for 7days. (**B**) Comparison of the viability rate of each group before and after the differentiation. (**C**) Neurospheres originating from SVZ/NSC. (Mean ± SEM; n = 3 independent experiments; *P < 0.05, **P < 0.001, one-way ANOVA), Scale bar: 50 *μ*m.

Using mirror and exposure of the cells to AgNPs has no significant impact on the efficiency of neurosphere (Fig. 5A,C).

### The viability of the cells before and after the differentiation

The number of live and dead cells in all three groups was counted before and after the differentiation using trypan blue. As shown in Fig. 5B, the cell viability percentage of the cells were in control (cell) = 85%, cell + mirror = 85%, cell + AgNPs = 80% before differentiation and were in cells = 75.6%, cells + mirror = 70%, cells + AgNPs = 72%, after differentiation. No significant difference was observed between the groups. But in individual groups, there was a significant reduction in viability rate of the cells after differentiation in cell and cells + mirror groups in comparison to before differentiation. There was no significant difference of viability rate before and after differentiation in cells + AgNPs group (Fig. 5B).

### UPE of NSCs in subculturing passages

**Figure 6:**
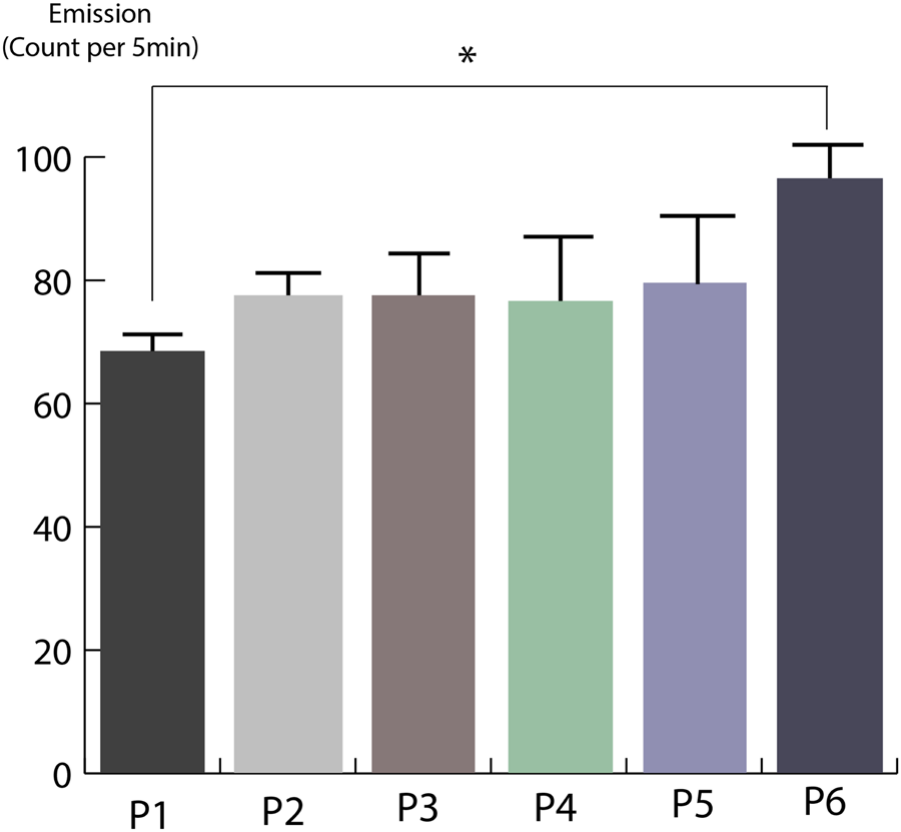
UPE from neural stem cells in subculturing passages. (Mean ± SEM; n = 3 independent experiments; *P < 0.05; ***P < 0.0001; one-way ANOVA). As we are using the relative UPE counts, the background counts of the detector and media are not shown.

The UPE intensity of the NSCs increased in serial passages in the control group, but only in the sixth subculturing passage (93 c.p.5 min) was significant in comparison to the primary passage (66 c.p.5 min) (*P* < 0.05). There was no significant difference between the UPE intensities of NSCs in the five serial subculturing passages (Fig. 6). It seems that the trend observed over the six subcultures reveals a pattern that is known to occur among protists^49,50^.

### UPE intensity of NSCs before and after the differentiation

**Figure 7:**
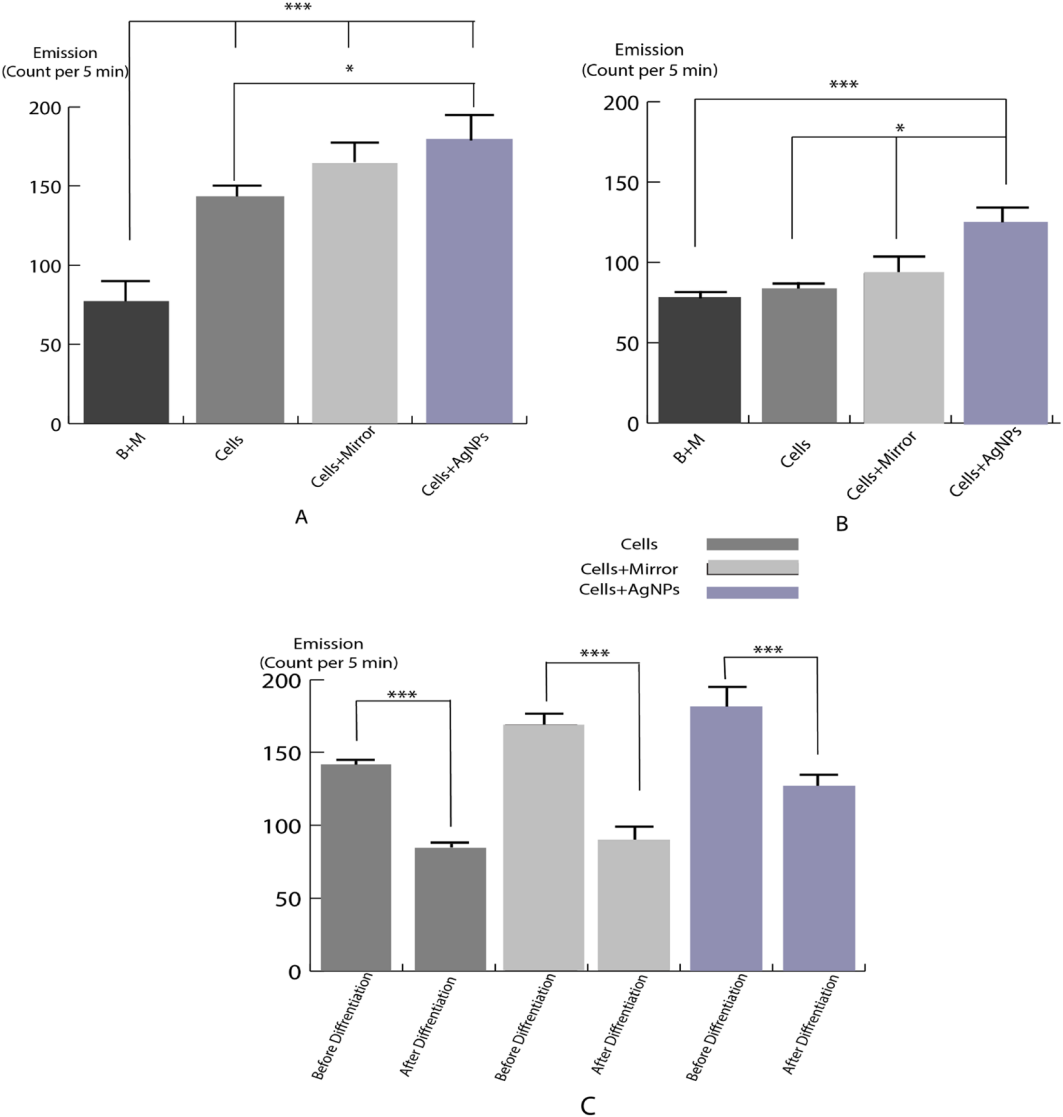
UPE intensity of “cells”, “cells + mirror” and “cells + Ag nanoparticles” groups before (**A** and **B**) after differentiation. (**C**) Comparison of the UPE of all individual groups before and after the differentiation. B + M = background and medium. (Mean ± SEM; n = 3 independent experiments; *P < 0.05; ***P < 0.0001; one-way ANOVA). As we are using the relative UPE counts, the background counts of the detector and media are not shown.

The results showed that before the differentiation, the UPE of NSCs in the cells as the control, cells + mirror and cells + AgNPs groups were significantly higher than the of background and medium (B + M) (77.67 c.p.5 min) (p < 0.0001). Moreover, the UPE of NSCs (i.e. exposed to AgNPs (178.7 c.p.5 min)) was significantly higher than of NSCs in the control group (139.7 c.p.5 min) (*P* < 0.05). No significant difference was observed in the UPE intensity of cells + mirror group (161 c.p.5 min) in comparison to control and AgNPs groups (Fig. 7A). Application of AgNPs in the culture of NSCs causes significantly increased UPE (116.3 c.p.5 min) relative to cell, cells + mirror (*P* < 0.05) and (B + M) groups after differentiation (*P* < 0.0001). There was no significant differences between control (83.33 c.p.5 min), cells + mirror (91.67 c.p.5 min) and (B + M) (77.67 c.p.5 min), groups (Fig. 7B). In fact, the UPE rates are low because of our subtracted dark count as well as low UPE detection at °C. This rates cannot be due to the cosmic ray noise, since cosmic rays always exist and we did not detect such rates when the detection was without samples. Comparison between the individual groups before and after the differentiation showed that the UPE intensity of cells after differentiation significantly lower than before the differentiation in each groups (*P* < 0.0001) (Fig. 7C).

### Differentiation capability of NSCs after exposure to mirror or AgNPs

**Figure 8:**
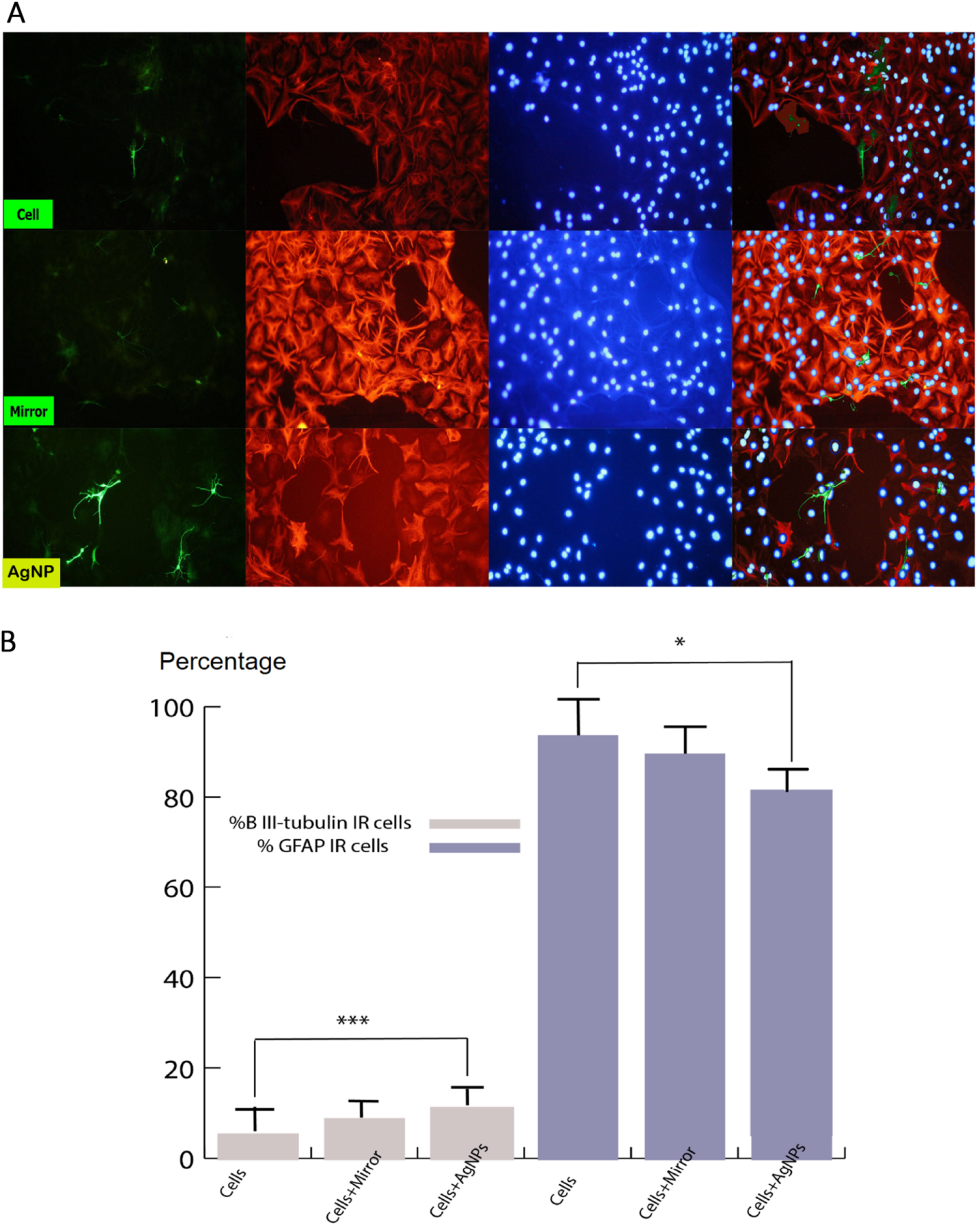
(**A**) Representative pictures of differentiated neural stem cells, for neuronal (*β*-III tubulin, green) and astrocyte (GFAP, red) Scale bars = 200 *μ*m (**B**) percentages of neuron, astrocyte after immunocytochemistry. IR = Immuno reactive, GFAP = glial fibrillary acidic protein. (Mean ± SEM; n = 3 independent experiments; *P < 0.05; ***P < 0.0001; one-way ANOVA).

Resulting cells from all three groups expressed *β*III-tubulin for the neuron, more glial fibrillay acidic protein (GFAP) for astrocyte after differentiation (Fig. 8A). Quantification of the number of astrocytes and neurons arising from treated and control cells showed that the percentage of neurons in the cells + AgNPs group (11.8%) was significantly higher than the control group (6.6%) (*P* < 0.0001). Moreover, the percentage of astrocytes in the cells + AgNPs group (80.2%) was significantly lower than of the control group (86.2%) (*P* < 0.05) (Fig. 8B).

## Discussion

This study describes the first demonstration of UPE measurement of neural stem cells that increases with serial sub-culturing passages, and also showed that the UPE intensity of the neural stem cells diminishes after the differentiation and increment of UPE by AgNPs more pushes the differentiation of the neural stems to the neuron.

### Neurosphere forming frequency

In this study, we investigated the effect of UPE on the neurosphere number and our results demonstrate that increasing of UPE by mirror and AgNPs have no effect on the number of neurospheres. Since exposing the cells to AgNPs and mirror took place on the 6th day, whereas the number of neurospheres have been counted a day later, it may not be a sufficient time for cellular communication and consequently formation of the neurospheres.

### UPE intensity and serial passages

As previous studies indicate that as the passage number increases in a cell culture, passage-dependent effects evolve including changes in expression of growth factors, messaging pathways, cell behaviors, cumulative cell damage and total changes in phenotype and genotype of the cell line^51^. It has been shown that the bone growth factors are influcencing UPE^52^ but we did not investigate the effect of grow factors on the UPE of NSCs directly. There is no specific and precise criterion for determining the number of passages required to make the cell line subject to wide variations. This number depends on the type of cell and the characteristics of the cell line^53^. No studies have ever been done on the changes that have taken place after each cell passages for neural stem cells. But as a general rule, as a cell ages and getting closer to death, one of the changes that occurs is the increase of free radicals which is in direct correlation to cellular UPE^54^. According to the results obtained in this study, the amount of UPE radiation increased in passages 1 to 6 cells, which is only significant between the sixth passage compared to the first passage, which probably indicates cellular changes from the sixth passage in neural stem cells.

### Previous studies on the effects of AgNPs on cells

Nanoparticles have very unique physical and chemical properties, which allow them to be used in numeorus areas like cosmetics, electronics, medicine, and pharmaceuticals, among them. Particle size and surface area of nanoparticles have key role in interaction of materials with biological processes^55^. Several studies demostrated that one of the major mechanisms for toxicity of the nanoparticles is due to the generation of oxidative mechanisms and free radicals i.e., perturbation of redox signal processes^56^. Perturbed or increased ROS formation could produce oxidative stress, inflammation and consequent damages to the proteins, cell membrane and DNA. It seems that size has an essential function since the smaller size is able to induce more ROS. However, nanoparticles have several beneficial properties, these also can produce serious hazard and toxic effects on cells. AgNPs are among the most widely used and investigated nanoparticles in diverse areas^57^ as well as in neurological researches^58^. For example, Kaur and Tikoo^59^ reported that the cytotoxicity effect of AgNPs depended on dose, surface potential of nanoparticles, and cells type in skin epithelial A431, lung epithelial A549 and murine macrophages RAW264.7 cells. Repar *et al*.^60^ examined the neurotoxicity induced by citrate-coated AgNPs (AgSCs) in neurons and astrocytes originated from human embryonic stem cells. When neurons and astrocytes were exposed to 0.1 *μ*g/mL of AgSCs the astrocyte/neuron ratio is increased and helped astrogenesis, but 5.0 *μ*g/mL of AgSCs significantly changed the morphology of astrocytes. In addition, AgSCs and AgNO_3_ produced different neuronal toxicity mechanisms. Furthermore, most of these effects were decreased when the cell culture was co-treated with AgSCs and the ascorbic acid, which suggest oxidative (redox) stress is the most important root of AgSC-produced astrocytic/neuronal toxicity and that antioxidants may present a neuroprotective outcome. Xu *et al*.^61^ studied AgNPs (20 nm) at various concentrations (1, 5, 10 and 50 *μ*g/mL) on primary rat cortical cell cultures. They revealed that 1–50 *μ*g/mL of AgNPs induced neuronal toxicity, degraded cytoskeleton components, perturbed pre- and postsynaptic proteins and mitochondrial processes that led to cell death. The fetus and neonates are particularly sensitive to the cytotoxicity. AgNPs perturbed early neuronal processes and increased neuron apoptosis through cellular oxidative stress and mitochondrial disruption in *in vitro* neural stem cells (NSCs) from fetal rat hippocampus^62^. Austin *et al*.^63^ investigated the effect of silver on pregnant mice and fetuses after intravenous injections of 10 nm AgNPs or soluble silver nitrate (AgNO_3_) that they found a remarkable silver accumulation in maternal liver, spleen and visceral yolk sac (VYS), which may have harmful effect on embryonic growth but insignificant in fetuses. In fact, despite beneficial effects and potential applications of nanoparticles such as stimulation of neuronal cell proliferation, axonal growth, neuronal cell adhesion, neuroprotection, and differentiation of stem cells into neuronal cells, they also have seversl harmful effects^58^ such as cytotoxic effects mainly via unregulated redox processes^57,58^. Perhaps, investigation of UPE from neural cells be a possible method elucidating the effets of various nanoparticles in neural cells.

### Ag nanoparticles, UPE intensity and cell viability

Present investigation showed that increased concentration of the AgNPs lead to reduced viability rate and UPE, suggested toxicity of AgNPs at high concentration for NSCs, which is in agreement with previous studies^63–65^. The present study also shows that the incubation of NSCs with AgNPs leads to 21.8% 28.3% increase in UPE intensity relative to the control group and 9.9% 21.2% to cells + mirror group, before and after differentiation, respectively. These findings are similar to those of previous reports in which AgNPs could rise the UPE intensity^45,46^. One of the more acceptable opinions about the source of UPE production is free radicals, mentioned as reactive oxygen species (ROS) and reactive nitrogen species (RNS)^66,67^. These species act as messenger molecules in the brain and are essential for messaging processes such as the release of neurotransmitters and the formation of memory^68–71^. Free radicals react sequentially with lipids or proteins and produce electron-excited species^72^. AgNPs play a role in stimulating UPE production through single oxygen production and another phenomenon called intrinsic fluorescence of the cell. It seems that metallic nanoparticle somehow transmits energy to adjacent molecules to make them excited^45,73^, depending on the time of being in the vicinity of cells, the type of cells, the radiation level and the size of nanoparticles^74^. Also, it would be interesting to identify a threshold emission level by which AgNPs induce autoluminescence, or how can a memory effect be induced once a “tolerable” threshold concentration of AgNPs has been exceeded by which the emissions no longer go back to background levels^75^. In addition, in order to understand the various pathways on how naoparticles interact with cells, see the reference^76^. In our research we have particularly used AgNPs while other nanoparticles can be investigated too, for example CuO-NPs are far more interesting in terms of toxicity, or the most widespread NPs are those using TiO_2_, and the most prominent candidate in terms of neuro-toxicity are Al-compounds that comodulate neurodegenerative diseases^77^. As a future prospect, it can be studied too whether our research can be connected to chronic atmospheric NP-exposure and translocation of ambient NPs via the BBB or the olfactory pathway directly into the brain^78^ along with the associated neuro-degenerative effects^79^? In theory such effects must also have an impact on the epigenome of NSCs^80^.

### Mirror and the auto-optic effect on cells

Mirrors act as reflectors of light. So far, there has no been a strict research on the effect of mirrors on UPE intensity. Petrash *et al*.^40^ measured spectrometrically the “emission” from the water and blood samples with different configuration of mirrors around the samples. Their results showed that the application of the mirror lead to more “emission” comparing with the control group without mirror. In our method, we intended to see if UPEs are shifted back to the sample by mirror^81^. Using the various materials as reflectors including gold, pure silver, and aluminum, it is expected that the most part of biophotn emission can be reflected toward the sample and this may play a role in enhancement of the UPE intensity. By using a small box made up of normal home mirrors, our results indicate that mirror slightly enhances the UPE intensity in the cells + mirror group, but it is not significant with regard to the control. The cause of this phenomenon may be related to the duration time and the nature of the mirror.

### UPE irradiation and differentiation

The intrinsic potential of NSC is differentiation into neuron and glial cells^82^. This process is regulated by genes and messenger paths. The PI3K/Akt messenger pathway plays an important role in the self-neuronal and neuronal activity of the neural stem cells located in the brain. It is possible that mitochondrial and ROS generation processes have key roles in AgNPs-induced neuronal differentiation achieved by the PI3K/Akt messenger pathway^83^, and the areas containing these cells have high levels of ROS^84^. Samberg *et al*.^85^ demonstrated that exposition of human adipose-derived stem cell to 10- and 20-nm AgNPs did not influence stem cell differentiation and did not produce significant cytotoxicity. However, here we observed a different result for differentiaion of NSC. Additionally, there is always the perception that active oxygen species have toxic effects on cells, but recent studies have shown that these species act as a second messenger^86^ during normal cellular processes. These species also influence the formation of neurospheres and differentiation of neural stem cells, so adding H_2_O_2_ to the cell, in addition to creating more neurospheres than the normal and similar, causes more differentiation of these cells into neurons^87^. On one hand, reactive species are a source of UPE production, and on the other hand the production of UPE can bring adjacent fluorophore molecules to the excited level and add to the number of these reactive species. Based on the results of the differentiation, in which the ratio of differentiation to the neuron is proportional to the increase in UPE, it can be analyzed that reactive oxygen species and UPEs are generated in each of the messaging paths that are in sync with each other. And these two are in a two-way relationship.

## Conclusion

In this paper, we have investigated the ultraweak photon emission from neural stem cells. We have observed that the intensity of UPE increased by serial passaging with significant enhancement on the sixth passage. The UPE intensity of stem cells after the differentiation were significantly lower than before the differentiation in each groups, which appropriated with the cell numbers. Then we studied whether the separate applications of mirror and AgNPs affect the UPE intensity and differentiation of stem cells. As a result, AgNPs significantly increased the UPE of the cells (compared to the control group) before and after the differentiation, but the effect of mirror was trivial on the UPE intensity before and after the differentiation. However, our results show that neither mirror nor AgNPs affect the neurosphere number, but treatment with AgNPs increased 44% neuronal differentiation of the harvested neural stem cells. Our results suggest that UPE measurement may be suitable for assessing and studying the effects of nanoparticles in living cells and neurons.
